# Laboratory investigation of steam transmission in unsaturated clayey soil under osmotic potential

**DOI:** 10.1016/j.mex.2017.06.003

**Published:** 2017-07-15

**Authors:** Mehdi Jalili, Mahmoud Nikkhah Shahmirzadi, Mahdi Kaseb Hazrati, Hamed Ahmadi

**Affiliations:** a Department of Civil Engineering, Technical & Engineering Faculty, Semnan Branch, Islamic Azad University, P.O. Box: 35135-179 Semnan, Iran; b Geotechnical Engineering, Department of Civil Engineering, Technical & Engineering Faculty, Semnan Branch, Islamic Azad University, Semnan, Iran

**Keywords:** Unsaturated clayey soil, Osmotic potential, Steam transmission, Dispersive clay

## Abstract

Liquids coming from different sources like wastewaters, agricultural and industrial activities and leakages of chemical substances often have high concentration of chemical compositions and the osmotic gradient generated around such sources causes a considerable transmission of the Contamination. The steam transmitted by non-polluted soils moves to polluted masses, causing an increase in the volume of pollution zone and movement of pollutants. Therefore, such physical and chemical processes should be taken into account in pollution transmission models. Using Crumb method, laboratory investigations were conducted on non-dispersive and dispersive clayey soil samples obtained from three areas in Zanjan Province of Iran. A simple experimental setup has been used and hereby introduced. The impact of osmotic force from salinities of 0.5, 1, and 1.5% on steam transmission in clayey soil was examined. Results indicate that for all samples between 5 to 15 days, the moisture content increased in the pollutant zone and decreased in the non-pollutant area. Also it was observed that for dispersive clayey soil, movement of steam among layers was observed to be orderly and its amount was higher than that of non-dispersive clayey soil.

## Highlights

• The impact of osmotic force from various salinities on steam transmission in non-dispersive and dispersive clayey soil examined.
• For all samples the moisture content increased in the pollutant zone and decreased in the non-pollutant area.
• For dispersive clayey soil, movement of steam among layers was orderly.

## Graphical abstract

**Figure:**
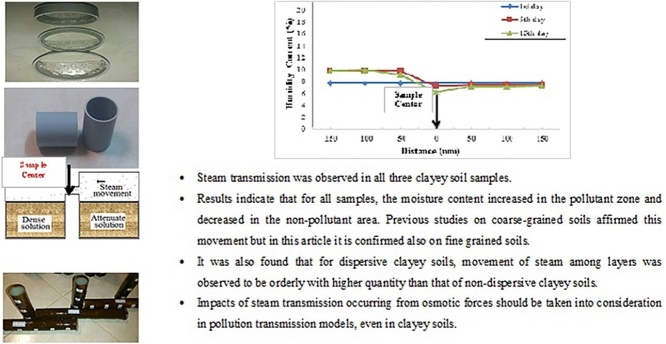

## Method details

In engineering sciences, soil is a non-integrated mix of various chemical compositions grains and decayed organic substances whose empty spaces are occupied by water, air (gases), and chemical pollutants. As an important construction material, soil is extensively employed in civil engineering and most structures are founded on soil. Therefore, civil engineers have to make a thorough study on soil properties such as origin of grading, water drainage capability, land subsidence, shear strength, compressibility, bearing capacity, steam transmission, etc. Transmission of steam in unsaturated soils is performed under the effect of thermal and osmotic gradient. Substances in the water available in soil pores bring about osmotic potential. Osmotic potential gradient is able to generate substantial water flow and steam in soils with high concentrations of chemical compositions. For water transmission to happen in soil through osmotic processes, a semi-permeable membrane is required to block passage of ions while freely permit water in.

In atmospheres with semi-permeable membrane properties, water under the impact of osmotic potential flows from area of low concentrations to areas of high concentrations. In the presence of such semi-permeable membrane, osmotic potential can cause a pressure difference in fluid. Steam transmitted from non-polluted soils to polluted masses by steam condensation above polluted and concentrated solutions may bring about an increase in volume of pollution zone and horizontal movement of pollutants in liquid form. Such physical and chemical processes, therefore, should be taken into consideration in pollution transmission models. It should be mentioned that the polluted zone means the zone with high concentration of the chemical compositions.

In recent years, geotechnical practice has increasingly encountered situations where the pore-fluid chemistry of soil varies under environmental impacts [1]. In some studies such as those performed by Gurr et al., Jury and Letey, Nassar et al. and Salzmann et al., steam transmission as a result of thermal gradient was investigated [2], [3], [4], [5]. In studies conducted by Goudarzi et al., steam transmission was examined in three soil types of silty clayey loam, loam, and sandy loam in unsaturated conditions at three salinity levels [6]. Results of their experiments showed that transmission of steam in light sandy soils and coarse-grained soils at all salinity levels are more and quicker than those in soils with heavier texture that contains more clay content.

Ma et al. proposed a conceptual constitutive model for unsaturated soils is to explain the influence of pore-fluid chemistry on the chemo mechanical behaviour of unsaturated clayey soils which is capable of addressing the effect of water content, concentration and species variation on the mechanical behaviour of the clayey soil [7]. Osmotic adjustment is a complementary strategy consisting in a decrease of plant osmotic potential through the accumulation of internal solutes without affecting cellular volumes [8]. This allows the plant to maintain a favourable water potential gradient and to absorb water for turgor maintenance [9].

The hormonal profile in relation with osmotic adjustment under salinity in Solanum lycopersicum and its halophyte wild relative Solanum chilense investigated and results suggested that the capacity to use inorganic ions as osmotic may improve salt resistance in Solanum chilense and those phytohormones could be involved in this process [10]. Salt stress has received substantial attention in recent years compared to other stresses, because it is progressively claiming arable land worldwide, hence threatening global agricultural production in the near future [11]. The experimental study was undertaken to examine the influence of salt stress on the vegetative growth of the plant by subjecting seedlings to 0, 25, 50, 100, 200 and 300 mM NaCl stress for 10 days. The plants were found to retain Na+ mainly in the root, with less affected leaf K+ concentration, and consequently very low shoot Na+/K+ ratios (<0.2) under all the stress treatments. The proline content significantly increased under the 100–300 mM treatments (18- to 244-fold), with a corresponding significant reduction in osmotic potential and hence high osmotic adjustment [11].

The above mentioned researches have been focused on the non-saturated soil/water and volatilization interactions as the chemistry and agricultural view of this subject. Some other researches have been done in order to evaluate the geotechnical aspects. The effect of different specimen preparation methods (compaction, reconstitution) on the hydro mechanical behaviour and microstructure of soil was studied through a series of soil-water retention tests by Sun and Zhou [12] and it has been found that the soil-water characteristic curves and residual gravimetric water content of compacted and reconstituted specimens are almost the same in the high suction range. So in the present study, it could be considered that the sample preparation had no effect on the results [12]. The investigation of the geotechnical behaviour of oil-contaminated soils was showed that the oil contamination decreased the liquid limit and plastic limit of the CL soil. Also contamination indicated a lower maximum dry density and optimum water content compared to uncontaminated soil. The unconfined compressive strength, (q_u_) was affected by the increase in oil content in contaminated soils [13].

Desiccation cracks diminish the strength and enhance the average hydraulic conductivity of a cracked soil mass. Thus, desiccation cracking is important in many geotechnical scenarios. Determination of the tensile strength during desiccation has been done by Varsei et al. [14]. Results are presented from desiccation tests performed on two clayey compacted soils of medium to high plasticity. The results demonstrate that the tensile strength of compacted soil is influenced by initial water content, suction, dry unit weight, soil pore structure, and soil type [14]. Thermal Conduction Heating (TCH) is a major component of In Situ Thermal Desorption (ISTD), a soil remediation technology in which heat and vacuum are applied simultaneously. As the soil is heated, water is boiled and Dense Non-Aqueous Phase Liquid (DNAPL) constituents in the soil are vaporized. The resulting steam and vapours are drawn toward extraction wells for in-situ and aboveground treatment. Compared to fluid injection processes, the conductive heating process is very uniform in its vertical and horizontal sweep. Field project experience at numerous TCH/ISTD sites has confirmed those maintaining target temperatures for several days results in extremely high destruction and removal efficiency of chlorinated volatile organic compounds (CVOCs), polychlorinated biphenyls (PCBs), polycyclic aromatic hydrocarbons (PAHs) and other DNAPLs. Despite high pre-treatment soil contaminant concentrations, post-treatment soil concentrations have typically been non-detect [15].

As described above, the steam transmission affects the both chemical and geotechnical properties of soils with various chemical compositions, so the present study intended to examine steam transmission quantity in three clay soils (non-dispersive and dispersive) in unsaturated conditions and under influence of osmotic pressure with different salinity levels using a simplified laboratory model. Two types of soils (non-dispersive and dispersive) evaluated in order to find the effect of this characteristic on the results.

## Materials and methods

### Materials

The soil samples used in this study were obtained from three locations in Zanjan Province of Iran; Gavazeng, Dandi and Tarom. In order to obtain better results and a real model, these studies were conducted on three types of clay soils, each being transformed to nine layers in the laboratory. According to performed studies and certain intentions pursued herein, the three lacations in Zanjan Province of Iran considered were chosen based on their capacities in pollution leakage and execution of important projects that include:

(a) Dandi non-dispersive clay,
(b) Gavazeng non-dispersive clay, and
(c) Tarom dispersive clay.

**Fig. 1:**
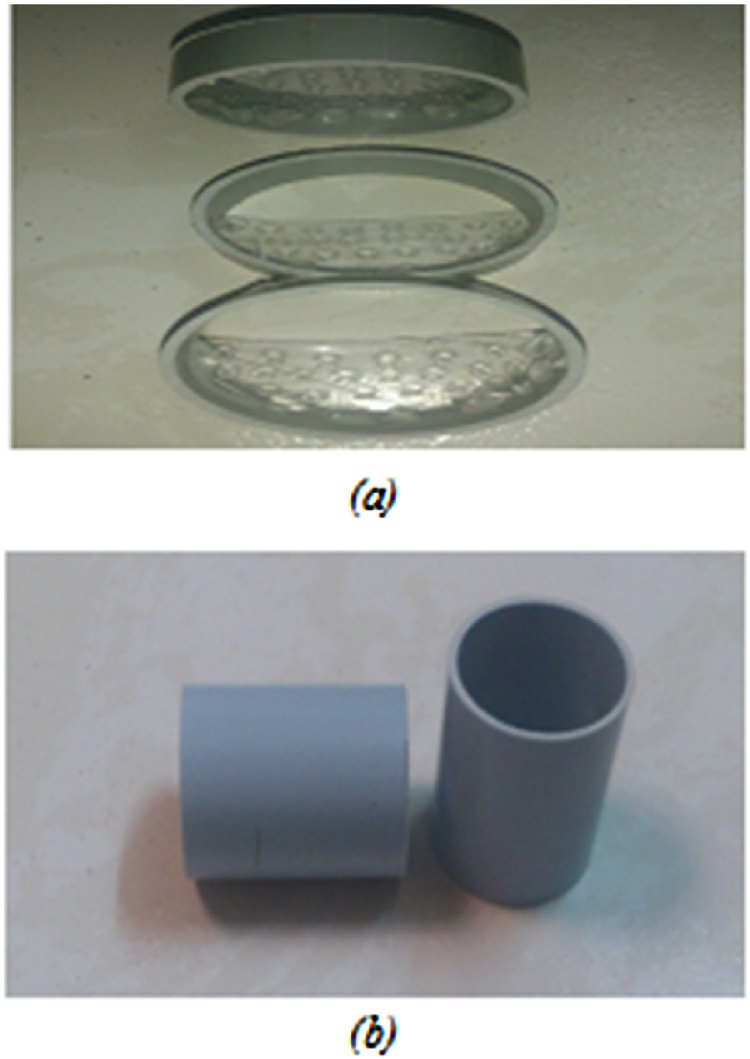
A view of rings with (a) perforated membrane and (b) layered parts.

Based on studies on different types of pipes, a polyethylene pipe was chosen. A length of 5 cm from the manufactured pipe (with 4 cm diameter and an appropriate length) was cut to be examined by profile machine. Then, edges are polished to form a cylindrical sample. This cut is made to prevent mixture of layered soils due to segmentation of 5 cm parts. This would facilitate cutting of two 5 cm parts from sticking position. To separate two concentrated and diluted environments, another environment named semi-permeable membrane was required. The length of this membrane was 1.5 cm with 4 cm diameter. Inside this ring, there is a perforated sheet to avoid mixture of the two environments’ soils upon experiments (see Fig. 1).

### Methods

#### Laboratory model

**Fig. 2:**
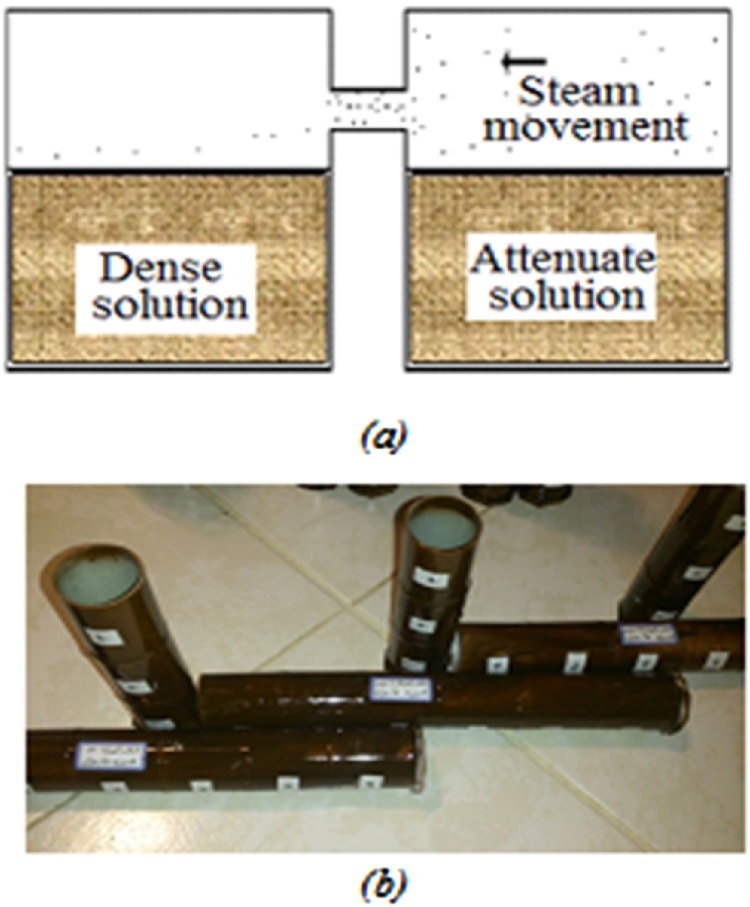
Steam transmission process from diluted to concentrated solution (a) schematic view and (b) constructed laboratory model.

When two concentrated and diluted solutions (or atmospheres) were placed side-by-side and related to one another, steam molecules are transmitted from the diluted environment to the concentrated one (see Fig. 2).

In order to examine the manner of the steam transmission and moisture content of soil in soil wedge, which could be responsible over extensive areas with different layers in horizontal direction of the larger model, a suitable model is required to present the behaviour of large soil masses at the area under study in smaller scales.

#### Development of concentrated and diluted environments

Concentrated (saline) and diluted (non-saline) environments are required to check the steam transmission. To do so, saline and non-saline areas were composed of 3 and 6 layers, respectively, which were connected to each other by a semi-permeable ring.

#### Determination of moisture content of samples

A percentage of moisture content was added to prepared clayey soil samples. This percentage was determined and measured by a pressure plate device at a suction of 3 atm. To investigate the influence of osmotic force on steam transmission, potential of samples’ primary matrix had, first of all, to be made equal at both saline and non-saline atmospheres. For this, moisture content percentage of soil samples at suction of 3 atmospheres was measured and in the next stage, in order to measure the transmitted moisture content, the equivalent moisture content was applied.

#### Moisture content applied to samples

Table A1 (see Appendix A) shows the maximum moisture content resisted by samples under pressure of 3 atmospheres in the pressure plate device. Given the laboratorial atmosphere, the difference between its physical conditions and outside space (nature), dispersity of sample 3 (Tarom clay), moisture content range was chosen between minimum and maximum points based on trial and error experiments (see Table A2 of the Appendix A).

#### Development of saline and non-saline sections

The Saline section segment of soil was humidified by normal NaCl solutions using the values presented in Table A2. Afterwards, it is kept within a sealed plastic in an insulated container for two days in order to achieve uniform moisture content. Salinity levels were applied based on dry weight of clay soil and the three salinity levels considered are 0.5, 1 and 1.5%.

Obtained moisture content was added to samples based on values presented in Table A2 to develop the non-saline section. These samples were kept within a sealed plastic in an insulated container for two days.

#### Measurement of transmitted steam

When both saline and non-saline sections of samples were prepared, they were immediately placed in developed tubes. Saline and non-saline sections were composed of three and six layers (sections), respectively. The two sections were adjacent to each other with a semi-permeable membrane between them while the set up was kept at a constant temperature of 21 °C. Incubator was used to keep temperature wholly constant, and prevent temperature influence on the steam pressure. Soil moisture content changes were monitored in both saline and non-saline sections in order to measure amount of transmitted steam. To achieve this, a portion of the moisture content was weighed after 5 and 15 days, whereby an entirely similar sample area was prepared for each timeline for each of the salinity level of 0.5, 1 and 1.5% and for the sample area to be placed in the incubator. After 5 and 15 days, samples were taken out of the device and 5 cm sections were immediately segregated to determine the amount of moisture content. This procedure was performed for the three salinity levels of considered and on the three clay soil samples.

## Results and discussions

Results showed that phenomenon of steam transmission in unsaturated soils under the influence of osmotic force depends on soil context and soil salinity levels. The results are in agreement with Scotter, Kelly and Selker and Weisbrod et al., [16], [17], [18]. In addition, results of this study indicated that the model presented by Kelly and Selker [18] has close relationship with the results of this study in low osmotic gradients. However, it shows inefficiencies in high osmotic gradients possibly due to the considerations taken for development of this model. Thus, it is proposed to conduct further simultaneous studies considering osmotic and thermal gradients. The general equation proposed by Kelly and Selker [18] is also suggested to be modified for better accuracy.

The results presented in Table A3 show the values of steam transmission in the three samples of clay soil categorized as non-dispersive (Dandi clay and Gavazeng clay) and dispersive (Tarom clay).

### Dispersive and non-dispersive clay soil

Soil dispersity occurs when repulsive forces among clay particles exceed attractive ones. In fact, particles repulse one another in the presence of water to perform a colloidal suspension. In non-dispersive soils, there is a speed limit, below which no erosion would take place. Individual particles stick to one another and are segregated only by water flow with erosive energy. For dispersive soils, however, there is no speed limit, and they are suspended even in the vicinity of still water. Therefore they are highly exposed to erosion and piping (establishment of erosion tunnels) even when the water is still. Dispersive soils have average-to-high clay content. Dispersive soils have higher sodium solution content (up to%12) in there pore water compared to ordinary soils. Clay particles act as connectors and cover around silt and gravel in soils with high concentrations of salt. The pH value of dispersive soils varies from 6 to 8. Simultaneously, the tension required for formation of erosion by clay chemical compositions, pH value, organic materials, heat, water, thixotropic and type and concentration of ions in pore and erosive liquid are influenced.

### Analysis of studies and observations

**Fig. 3:**
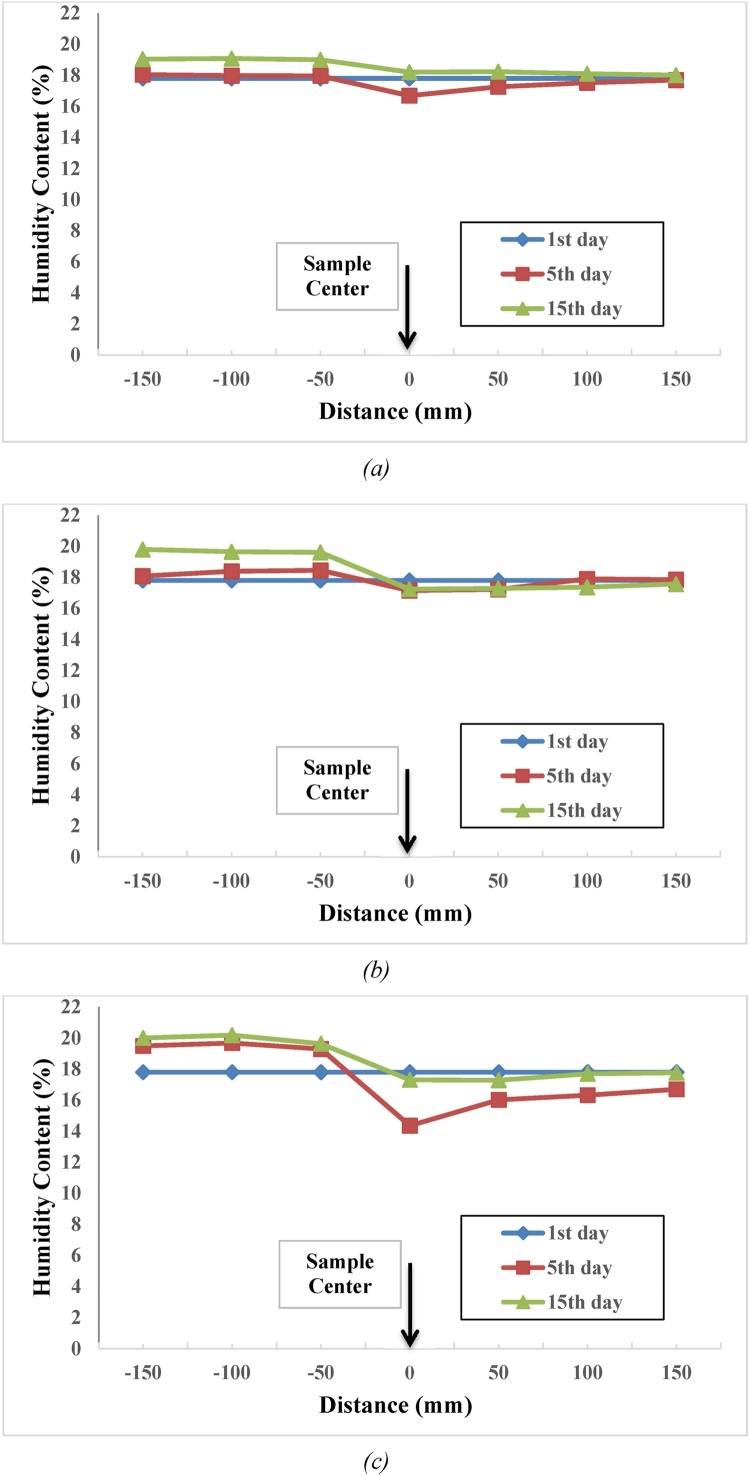
Distribution of moisture content in laboratory model for Dandi clay soil with salinity level of (a) 0.5, (b) 1, and (c) 1.5%.

Results of 5-day salinity test for the 0.5, 1 and 1.5% salinity levels obtained from Dandi clay are shown in Fig. 3. Due to shortness of the time interval within which steam was transmitted from the first layers of the non-saline area (layers far from saline environment) to saline area (layers close to saline environment) of the setup, the layer adjacent to the non-saline environment attracted the highest steam. On the other hand, results of 15-day salinity test indicated that the process of steam transmission from first layers of non-saline area to saline area takes more than five days. In saline area, the last layer attracts the highest amount of steam which may be due to longer time of the test. The higher the soil’s salinity level, the quicker the steam transmission process and the better the concentrated environment would be in terms of moisture content attraction. In an experiment conducted using Crumb method, Dandi clay soil is of non-dispersive type. Plaster grains were found in this area’s clay soil. Addition of watery plaster to soil brings about integration of clay particles and reduction of disparity potential. Its action is the replacement of sodium ions by calcium ions. As plaster is a relatively less soluble substance in water and due to effect of plaster, some discordance may be raised in comparison to other tests. Steam transmission from a diluted to a concentrated environment has a slow speed, and existence of plaster and clay soil’s non-dispersive properties is the main cause for disorderliness of the results as presented in Fig. 3.

**Fig. 4:**
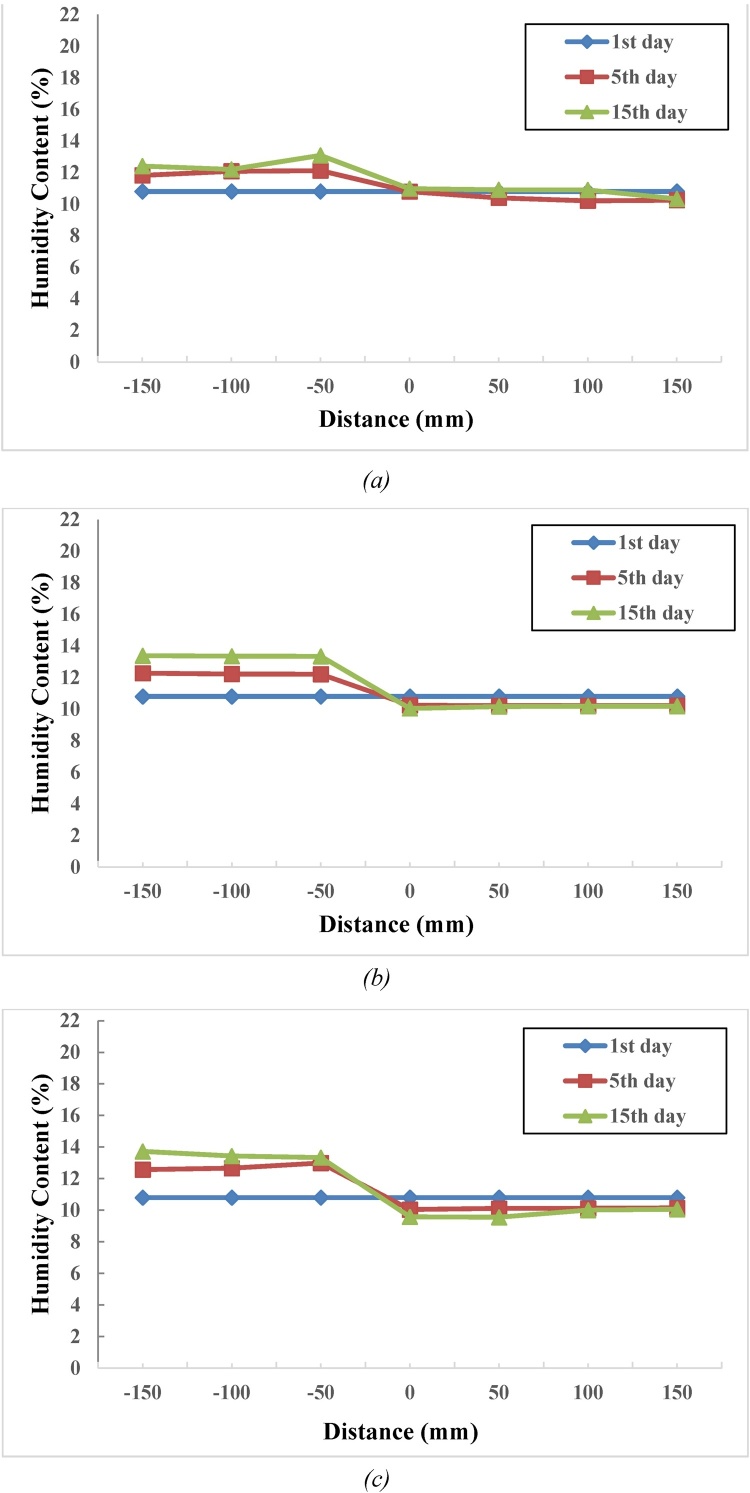
Distribution of moisture content in laboratory model for Gavazang clay soil with salinity level of (a) 0.5, (b) 1, and (c) 1.5%.

Results of 5-day salinity tests at 0.5, 1 and 1.5% salinity levels conducted on *Gavazeng Area clay soil* are presented in Fig. 4. In saline area, the layer next to the non-saline area has the highest amount of steam attraction. Fig. 4 also illustrates 15-day salinity test. Due to longer time allocated to the test, steam transmission process from the first layers of non-saline area to saline area takes more than five days, and in saline area, the last layer has attracted the highest amount of steam. The higher the soil’s salinity level, the quicker the steam transmission process and the better the concentrated environment would be as moisture content attractor. According to Crumb test, clay soil of Gavazeng Area is non-dispersive, in which, unlike Dandi clay soil, plaster particles are inexistent, and steam transmission procedure from diluted to concentrated environments is disordered (see Fig. 4).

**Fig. 5:**
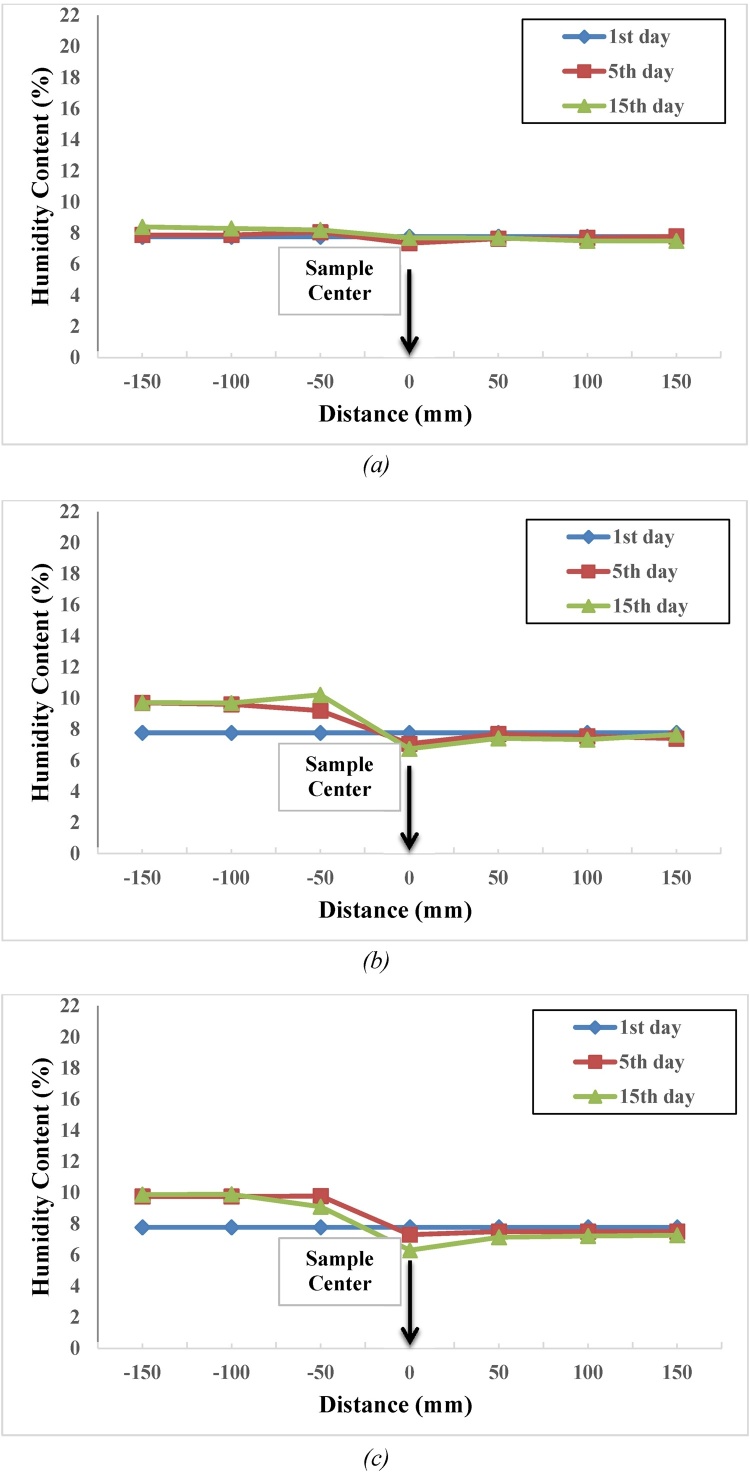
Distribution of moisture content in laboratory model for Tarom clay soil with salinity level of (a) 0.5, (b) 1, and (c) 1.5%.

Results of 1, 5 and 15 days salinity test at 0.5, 1 and 1.5% salinity levels obtained from Tarom clay soil are shown in Fig. 5. Results of 15-day salinity tests indicated that the process for steam transmission from first layers of non-saline area to saline area takes more than five days. In saline area, the last layer attracts the highest amount of steam. It was observed that the clay soil sample is of dispersive type. The process for steam transmission from diluted to concentrated environments was orderly and the steam movement factor as well as attraction inclination in concentrated environment may be attributed to dispersity of this area’s clay soil properties (see Fig. 5).

## Conclusions

In this article, steam transmission in three clay soil types of non-dispersive (Dandi and Gavazeng Area) and dispersive (Tarom) in Zanjan Province of Iran in unsaturated conditions and at three salinity levels of 0.5, 1, and 1.5% were examined. Based on observations on the experiment conducted on the soil samples and since clay soil is composed of heavy textures, steam transmission was confirmed in all the three samples. It was observed that the higher the salinity level, the higher the steam transmission quantity from non-saline to saline areas. Results obtained from clay soil tests significantly show that in case of heavy clay soils, if osmosis gradient levels existing in air gaps (fine fissures among soil ingredients) are increased, then system equilibrium would sooner occur and steam transmission will stop. Soil water chemical compositions gave rise to generation of osmosis potential gradient, causing a considerable steam transmission amount. Therefore, in adjacent to polluted resources such as chemicals, soluble oils, radioactive waste reservoirs, landfills, injection of hazardous wastes, agricultural practices, etc., which often have high salt concentrations and generate very high osmotic gradients, steam transmission from non-polluted soils to polluted masses causes an increase in pollution volume and horizontal movement of pollutants in liquid form. That is why effect of steam transmission due to osmotic forces should be taken into consideration in pollution transmission models.

## Figures and Tables

**Table A1:** Results of moisture content obtained by pressure plate device.

Percentage of moisture content at pressure of 3 atm for three clayey samples				
Sample	Container weight	Weight of container and mud	Weight of container and dried soil	Percentage of moisture content
Dandi I clay	30.24	51.07	46.83	25.56
Dandi II clay	32.45	51.77	47.86	25.37
Gavazeng I clay	42.49	85.21	81.19	10.38
Gavazeng II clay	41.34	75.88	72.66	10.28
Tarom I clay	39.81	62.89	57.31	31.86
Tarom II clay	31.43	53.97	48.59	31.35

**Table A2:** Amount of moisture content applied to samples.

No.	Sampling place	Applied moisture content
1	Dandi clay	θm = 17.8
2	Gavazeng clay	θm = 10.8
3	Tarom clay	θm = 7.77

**Table A3:** Results obtained from experiment of non-dispersive (Dandi and Gavazeng) and dispersive (Tarom) clay soils.

Salinity (%)	Time (day)	Sample no.	Non-saline area						Saline area		
			1st layer	2nd layer	3rd layer	4th layer	5th layer	6th layer	1st layer	2nd layer	3rd layer
0.5	5	I	17.9	17.8	17.7	17.5	17.3	16.7	18.0	18.0	18.0
		II	0.3	10.3	10.3	10.2	10.4	10.8	12.1	12.1	11.8
		III	7.8	7.8	7.8	7.7	7.6	7.4	8.1	7.9	7.9
	15	I	17.8	17.9	18.0	18.1	18.2	18.2	19.0	19.1	19.0
		II	10.3	10.3	10.3	10.9	10.9	11.0	13.1	12.2	12.4
		III	7.5	7.5	7.5	7.5	7.7	7.7	8.2	8.3	8.4
1	5	I	17.9	17.9	17.9	17.9	17.2	17.2	18.5	18.4	18.1
		II	10.3	10.3	10.2	10.2	10.2	10.2	12.2	12.2	12.3
		III	7.3	7.5	7.4	7.6	7.7	7.1	9.2	9.6	9.7
	15	I	17.8	17.8	17.6	17.4	17.3	17.2	19.6	19.7	19.8
		II	10.2	10.2	10.2	10.2	10.2	10.0	13.3	13.4	13.4
		III	7.8	7.7	7.7	7.3	7.4	6.7	10.2	9.7	9.7
1.5	5	I	17.1	16.9	16.7	16.3	16.0	14.4	19.3	19.7	19.5
		II	10.2	10.2	10.1	10.1	10.1	10.0	13.0	12.7	12.6
		III	7.6	7.6	7.5	7.5	7.5	7.3	9.8	9.8	9.8
	15	I	17.9	17.9	17.8	17.7	17.3	17.3	19.6	20.2	20.0
		II	10.3	10.2	10.1	10.0	9.6	9.6	13.3	13.4	13.7
		III	7.6	7.4	7.3	7.2	7.1	6.3	9.1	9.9	9.9
